# Antihelminthic Activity of *Lophira Lanceolata* on *Heligmosomoides polygyrus* Using an Automated High-Throughput Method

**DOI:** 10.1155/2023/9504296

**Published:** 2023-01-20

**Authors:** Yamssi Cédric, Noumedem Anangmo Christelle Nadia, Simeni Njonnou Sylvain Raoul, Samuel Berinyuy, Mounvera Abdel Azizi, Tientcheu Noutong Jemimah Sandra, Ngouyamsa Nsapkain Aboubakar Sidiki, Vincent Khan Payne

**Affiliations:** ^1^Department of Biomedical Sciences, Faculty of Health Sciences, University of Bamenda, P.O. Box 39, Bambili, Cameroon; ^2^Department of Microbiology, Haematology and Immunology Faculty of Medicine and Pharmaceutical Sciences, University of Dschang, P.O. Box 96, Dschang, Cameroon; ^3^Department of Internal Medicine and Specialties, Faculty of Medicine and Pharmaceutical Sciences, University of Dschang, P.O. Box 96, Dschang, Cameroon; ^4^Department of Medical Laboratory Sciences, Faculty of Health Sciences, University of Bamenda, P.O. Box 39, Bambili, Cameroon; ^5^Department of Animal Biology, Faculty of Science, University of Dschang, P.O. Box 067, Dschang, Cameroon

## Abstract

**Background:**

There are about 13 parasitic infections that are responsible for significant morbidity and mortality but have not received the attention they deserve; thus, they are now known as “neglected tropical diseases” (NTDs). This study was aimed at evaluating the antihelminthic activities of *Lophira lanceolata* using an automated high-throughput method.

**Methods:**

The antihelminthic activity effect of the extracts against *H*. *polygyrus* was determined using an automated high-throughput method. For the egg-hatching test, 100 *μ*L of embryonated egg suspension (60 eggs) was added to 100 *μ*L of various concentrations of extracts, levamisole, and 1.5% DMSO in a 96-well round-bottom microtitre plate. After mixing, the 96-well microplate was placed in WMicroTracker and incubated for 24 h at 25°C; the movements were recorded every 30 minutes. The same procedure was used for the larval motility assays, where 100 *μ*L of L1 or L2 larvae (50 larvae) were put in contact with 100 *μ*L of various concentrations of extracts.

**Results:**

The ovicidal activity (hatching) had an IC_50_ of 1.4 mg/mL for the ethanol extract. The aqueous and ethanol extracts of *L*. *lanceolata* showed larvicidal activity on the L1 larvae with IC_50_ of 1.85 mg/mL and 2.4 mg/mL, respectively, as well as on the L2 larvae with IC_50_ values of 1.08 mg/mL and 1.02 mg/mL for the aqueous and ethanol extracts, respectively. These results showed that the aqueous extract exhibited a stronger inhibitory power on the hatching rate of parasites than ethanol extracts, while the contrary effect was observed for the larval motility assays.

**Conclusion:**

This study provides scientific data on the use of *L*. *lanceolata* by the local population for the treatment of helminthiases. However, *in vivo* and toxicity tests are necessary to assess its activity and safety.

## 1. Introduction

Helminthiasis is one of the most devastating diseases in most developing countries in Africa. Most of the research funding for the control of diseases is directed toward HIV and tuberculosis [1]. Little or no attention is paid to helminth infections; hence, they are called neglected tropical diseases (NTDs). According to a recent study conducted by Cedric et al. [2], a prevalence of 23.5% was observed amongst children in Bambili, Northwest Region of Cameroon. Similar observations were performed by Igore et al. [3], where they had observed a prevalence of 26.4% in Tonga subdivision, West Region of Cameroon. All these high prevalence rates indicate that Cameroon has a serious problem of helminthiases.

Most of the helminth parasites are showing resistance to available synthetic drugs. Furthermore, these drugs have side effects and are not easily available to the local population [4]. In Cameroon, the use of herbal medicines for the treatment of several ailments including helminthiases is very common [5]. The local population of the west region of Cameroon usually relies on medicinal plants to overcome this problem of resistance, availability, and high cost of these synthetic drugs. Resistance to antihelminthic drugs is on the rise, in part due to their injudicious use [6]. It is therefore of paramount importance to rapidly screen medicinal plants used by the local population to treat helminthiases in order to justify their usage by the local population. Furthermore, little is known about the optimum dose and treatment frequency of such plants, as well as potential toxicities. Therefore, alternative control strategies are urgently needed to validate these medicinal plants.

*Lophira lanceolata* is a medicinal plant used in traditional medicine to treat many diseases. The roots are used to treat wounds, amenorrhoea, sterility, constipation, diarrhoea, jaundice, and vomiting [7]. The bark is used to treat kwashiorkor, jaundice, acute hepatitis, enteritis, coughs, and bronchitis [8]. The twigs are used to treat gingivitis, hypertension, dysentery, and syphilis. In the dry regions of West Africa, the oil extracted from the seeds is used as a body balm against lice and leprosy. The oil is used for many medico-religious purposes [9]. *Lophira lanceolata* is used by the local population of the western region of Cameroon for the treatment of helminthiasis.

Most conventional *in vitro* screening methods for parasitic worms have had limitations in that most of them are time-consuming to perform and/or lack repeatability/reproducibility [10].

The WMicroTracker (Phylumtech, Argentina), is a rapid, automated high-throughput equipment used for the evaluation of the antihelminthic activity of drugs by sending infrared beams to worms and recording the movement of worms [11]. *Heligmosomoides polygyrus* is a parasitic nematode (as opposed to nonparasitic nematodes such as *Caenorhabditis elegans*) used as a model for the evaluation of the antihelminthic activity of drugs. Studies carried out using *H*. *polygyrus* in this domain include those of Teufack et al. [12], Poné et al. [13], and Komtangi et al. [14]. This paper shows for the first time that *H*. *polygyrus* is used with the WMicroTracker system for the evaluation of the antihelminthic activity of a medicinal plant. The aim of this study was to evaluate the antihelminthic activity of *Lophira lanceolata*, a medicinal plant used for the treatment of helminthiasis in Foumban, West Region of Cameroon, and provide scientific evidence on its use as an antihelminthic agent.

## 2. Materials and Methods

### 2.1. Helminth Parasite

The infective third-stage larvae (L3) of *H*. *polygyrus* were generously provided by Pr. Rick Maizels, University of Edinburgh, UK. The parasite was cultured from the egg to the L2 stage in Petri dishes containing wet filter paper. It is a strongylid nematode related to human hookworm species. *Heligmosomoides polygyrus* is a standard experimental model used for routine screening of potential drug candidates [15].

### 2.2. Collection and Identification of Plant Materials

The leaves of *Lophira lanceolata* were harvested from the peripheral Savannas of Foumban in the Noun Division of the West Region of Cameroon. The plant leaves, flowers, and fruits were sent to the National Herbarium of Cameroon (NHC) for authentification, and it was registered and given the registration number 3512/SRFK-CAM.

### 2.3. Preparation of Extracts

The aqueous extract was prepared by infusion, while the ethanol extract was obtained using the procedure described by Poné et al. [13]. Briefly, 100 g of powder was weighed using an electronic balance (SF-400) and introduced into a 5 L container. One liter (1 L) of 95% ethanol was added, and the mixture was stirred for 5 minutes. The mixture was macerated for 72 hours at room temperature, and every 24 hours, this mixture was stirred. The homogenate was first filtered using a 150 *μ*m sieve and then with Whatman paper N°1. The filtrate obtained was introduced into an oven at 40°C for evaporation until the dry extract was obtained. The same procedure was performed for infusion except that water was heated at 100°C and that the powder was added.

### 2.4. Collection and Concentration of *H*. *polygyrus* Eggs

Freshly passed out faeces of monoinfected mice (*Mus musculus*) with a resistant helminth (*H*. *polygyrus*) was collected using teaspoon. 5 g of faeces was homogenized in a mortar using a pestle with 60 mL of saturated NaCl solution. This solution was successively filtered using a tea sieve and then a 150 *μ*m sieve. The filtrate was used to obtain *H*. *polygyrus* eggs through the flotation technique. The eggs were centrifuged three times for 10 minutes at 1500 rpm, which enabled us to wash all salt from the eggs. After centrifuging for 10 minutes, the solution was then siphoned using a pipette, distilled water was added, and the solution was centrifuged again.

### 2.5. Coproculture and Collection of *H*. *polygyrus* Larvae

The eggs obtained above were cultured by incubating them in a Petri dish. The Petri dish was incubated at 25°C at a relative humidity (RH) of 78% for 2 and 4 days, respectively, with the aim of obtaining L1 and L2 larvae.

### 2.6. Egg-Hatching Test

The antihelminthic activity of the extract was determined using the method described by Poné et al. [13] with slight modifications. Briefly, 100 *μ*L of the embryonated egg suspension containing 60 eggs was incubated with 100 *μ*L of various concentrations of extracts, levamisole, and 3% DMSO in 96-well cell culture round-bottom microtitre plates. The round-bottom microtitre plates were used so that WMicroTracker could directly send laser beams to worms. The final concentrations in the test plates varied from 5 to 0.3125 g/mL (1.5% DMSO) for the extracts and 5 *μ*g/mL for levamisole in a final volume of 200 *μ*L. The 96-well microplates were incubated for 24 h at 25°C at a relative humidity (RH) of 78% in WMicroTracker, where the worm movements were measured by using WMicroTracker every 30 minutes. Three independent experiments for the ethanol and aqueous extracts were carried out, and the average results were recorded. The inhibition percentages of hatching of each sample were determined at the end of incubation, using the average movement over 24 h in the presence of test samples with the extract, compared with the controls, DMSO 1.5% and levamisole [16]:

### 2.7. Larval Motility Assays

For the effects of the extracts on L1 and L2 larvae, the same procedure described above was followed. Briefly, 100 *μ*L of the L1 larval stage suspension containing 50 L1 larvae was incubated with 100 *μ*L of various concentrations of extracts, levamisole, and 1.5% DMSO in 96-well cell culture round-bottom microtitre plates. The 96-well microtitre plates were incubated in WMicroTracker at 25°C for 24 h; the worm movements were recorded every 30 min. The larval motility activity was determined by calculating the inhibition percentages [6]. The same method was used to test the activity of active compounds on the L2 larvae.

### 2.8. Phytochemical Screening

The extracts were tested for the presence of sterols, alkaloids, triterpenoids, saponins, anthocyanins, and anthraquinones using standard procedures described by Harbone [17].

### 2.9. Ethical Consideration

Ethical approval (N°2022/0032H/UBa/IRB) was obtained from the Institutional Review Board at the Faculty of Health Science of the University of Bamenda, by following the standard ethical guidelines for the use and care of laboratory animals (guidelines of the European Community; EEC Directive 86/609/EEC, November 24, 1986).

### 2.10. Statistical Analysis

The data were analysed by entering the values into a statistical package called GraphPad Prism version 8.0 for Windows (GraphPad Software Inc., San Diego, CA, USA). One-way ANOVA and Tukey's multiple comparison test were used to assess the antihelminthic studies. 50% inhibitory concentrations (IC_50_) were determined using the concentration-response curves obtained by plotting the logarithm of the concentration as a function of percentage inhibition.

## 3. Results

### 3.1. Egg Hatch Assays

**Figure 1:**
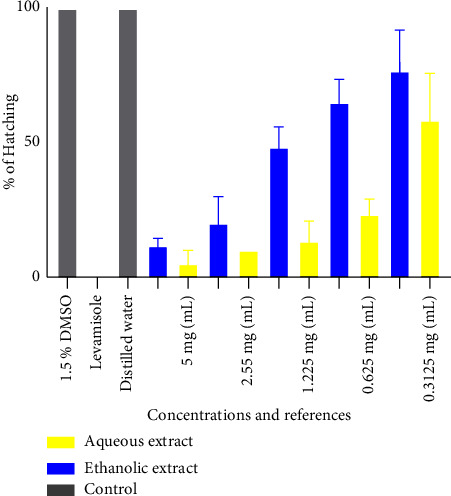
Effect of the aqueous and ethanol extracts on the percentage of hatching.

Figure 1 shows the percentage of hatching of the aqueous and ethanol extracts. It follows from the analysis of the figure that hatching was significantly inhibited at 5 mg/mL where we observed 5.0 ± 5.0% and 11.66 ± 2.8% for the aqueous and ethanol extracts, respectively. Distilled water and 1.5% DMSO had 100% hatching, indicating that the plant extract had an effect. The highest percentage of hatching for the extracts was observed with the ethanol extract at concentrations of 0.3125 mg/mL and 0.625 mg/mL with hatching percentages of 65 ± 8.6% and 76.66 ± 15.2%, respectively.

**Figure 2:**
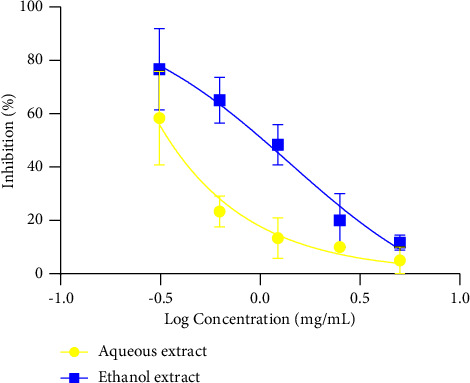
Inhibitory concentration 50 (IC_50_) of egg hatching.

Figure 2 shows the IC_50_ of the different extracts on the hatching rates of *H*. *polygyrus*. It follows from the analysis of the figure that the IC_50_ for the ethanol extract was 1.4 mg/mL and that for aqueous was undetermined because it was very wide, showing that the ethanol extract exhibited a stronger inhibitory power on the hatching rate of parasites.

### 3.2. Larval Motility Assays

**Figure 3:**
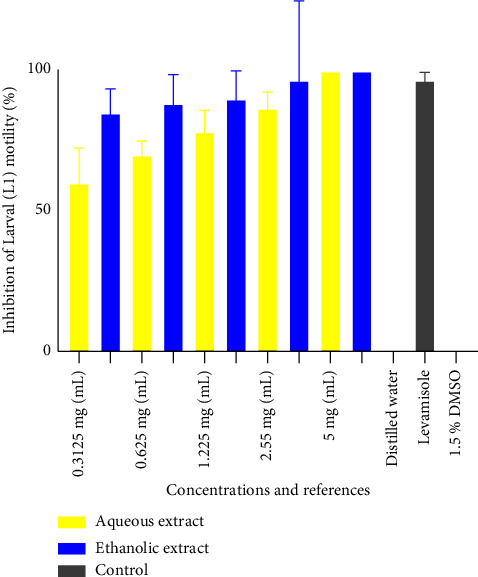
Inhibition percentage of the aqueous and ethanol extracts on L1 larvae of *H*. *polygyrus*.

Figure 3 shows the inhibition percentage of the aqueous and ethanol extracts on L1 larvae of *H*. *polygyrus*. It appears from the figure that the mean inhibition rate at 5 mg/mL completely inhibited (100%) the mobility of the L1 larvae as compared to the positive control (levamisole) that had a mean inhibition rate of 96.67 ± 2.88. The ethanol and aqueous extracts had a mean inhibition rate of 96.66 ± 6.44 and 86.66 ± 5.77 at 2.5 mg/mL, respectively. Both distilled water and 1.5% DMSO (negative control) had the mean inhibition percentages of 0%, indicating that the negative controls had no inhibitory effect on the larvae. The ethanol extract showed a higher inhibitory effect than aqueous extracts. Both the extracts showed a significant concentration-dependent effect. Their effects decreased with a decrease in concentration from 5 mg/mL to 0.3125 mg/mL.

**Figure 4:**
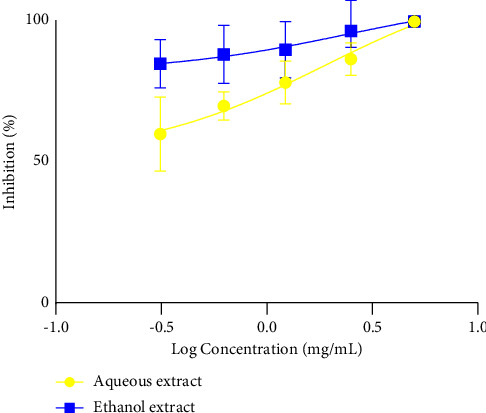
First larval stage, larval motility IC_50_.

Figure 4 shows the IC_50_ of the different extracts on the first larval stage of *H*. *polygyrus*. From the analysis of the figure, the IC_50_ for both the extracts was 1.85 mg/mL and 2.4 mg/mL, respectively. It follows from the analysis of the IC_50_ that the ethanol extract was more active than the aqueous extract.

**Figure 5:**
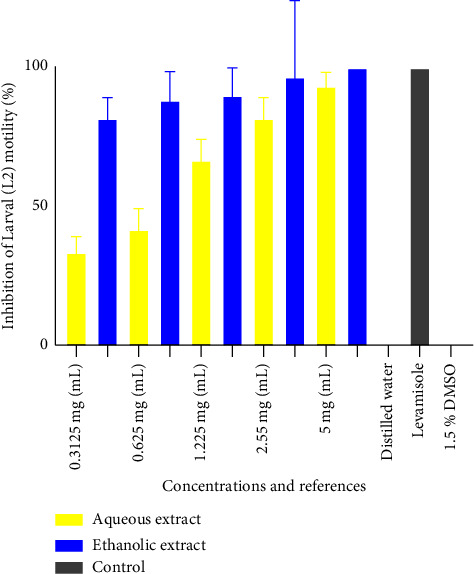
Inhibition percentage of the aqueous and ethanol extracts on L2 larvae of *H*. *polygyrus*.

Figure 5 shows the inhibition percentage of the aqueous and ethanol extracts on L2 larvae of *H*. *polygyrus*. It follows from the analysis of the figure that concentration 5 mg/mL had the highest mean inhibition rate of 93.33 ± 5.77 and 100 ± 0.0, respectively, for the aqueous and ethanol extracts, with a significant difference. The aqueous and ethanol extracts at the same concentration inhibited the L2 larvae with a significant difference. Distilled water and 1.5% DMSO (negative control) had no effects, while levamisole had 100% inhibition. The lowest inhibitory activity was observed at 0.3125 mg/mL with inhibitory percentages of 33.33 ± 3.21% and 90 ± 10% for the aqueous and ethanol extracts, respectively.

**Figure 6:**
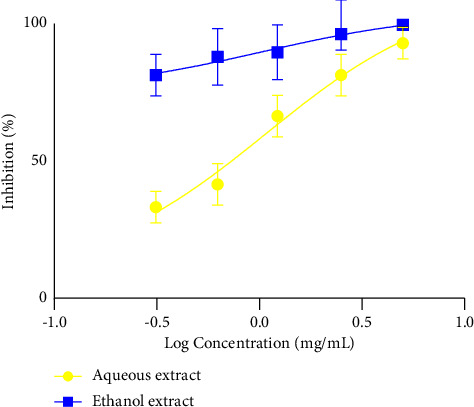
Second larval stage, larval motility, IC_50_.

Figure 6 shows the IC_50_ of the different extracts on the second larval stage of *H*.*polygyrus*. The analysis indicated that the IC_50_ for the aqueous extract was 1.08 mg/mL, while that for the ethanol extract was 1.02 mg/mL, indicating that the ethanol extract was more active than the aqueous extract.

Table 1 shows the phytochemical screening of *Lophira lanceolata* extracts. It follows from the analysis of the table that the aqueous extract contains more classes of chemical constituents.

## 4. Discussion

This study aims to evaluate the antihelminthic activity of *L*. *lanceolata*, a medicinal plant used for the treatment of helminthiasis in Foumban, West Region of Cameroon, and provide scientific evidence on its use as an antihelminthic agent.

The ovicidal activity (hatching) had an IC_50_ of 1.4 mg/mL for the ethanol extract. These results show that the ethanol extract was more active than the aqueous extract. Similar finding was obtained by Poné [13] with extracts of *Ageratum conyzoides* on embryonated eggs of *H*. *bakeri*. Egg hatching could have been inhibited by saponins in the extracts, as these molecules are known to stop nematodes from egg hatching [18]. The plant extracts inhibit the development of structures required for hatching in *H*. *Bakeri* such as a protractible stylet mouthpart that is used for both infection and opening of the eggshell during hatching [19]. Moreover, the plant extract may have stopped the exchange of water and sugars across the eggshell, altering the osmotic pressure and the size of the egg, thereby inhibiting the hatching process [20].

The aqueous and ethanol extracts of *L*. *lanceolata* showed larvicidal activity on the L1 larvae with an IC_50_ of 0.209 ± 0.05 mg/mL and 0.0033 ± 0.0060 mg/mL, respectively, as well as on the L2 larvae with an IC_50_ of 1.85 mg/mL and 2.4 mg/mL, respectively, as well as on the L2 larvae with IC_50_ values of 1.08 mg/mL and 1.02 mg/mL, respectively. The controls (distilled water and 1.5% DMSO) did not kill the larvae. The extracts of *L*. *lanceolata* possessed larvicidal properties, with the ethanol extract being the most effective one. Similar results were obtained by Kalmobé et al. [21], where the ethanol and the MeOH-CH_2_Cl_2_ extracts of the leaves of *L*. *lanceolata* showed activity with higher IC_50_ values on *C*. *elegans *wild-type strains compared to ivermectin, levamisole, and albendazole after 72 h of incubation.

This confirms the fact that the dead larvae found inside the treated 96-well microplates were due to the active ingredients of the plant extracts, thus indicating their larvicidal properties. This observation was also reported by Gertrude et al. [22] when evaluating *in vitro* ovicidal and larvicidal activities of the aqueous and ethanol extracts of the leaves of *Bidens pilosa* (Asteraceae) on *Heligmosomoides bakeri* (nematoda: Heligmosomatidae).

Throughout this study, we observed that L1 larvae were more vulnerable to different plant extracts than L2 larvae. The larvicidal properties of these extracts may be due to the penetration of active compounds across the cuticle of the larvae on one hand or the absorption of the substance by the larvae. This finding is similar to that obtained by Payne [23]. Payne [23] mentioned that active compounds penetrate the cuticle of nematodes and prevent the absorption of glucose or block postsynaptic receptors, thus paralyzing the larvae. The active compound may inhibit the transmission of nervous impulses by gamma-aminobutyric acid (GABA) whose secretion is stimulated by the active compound [23]. Lem et al. [24] mentioned that active compounds found in food can cross the intestinal lining of larvae and gain access to the circulatory system of the organism. Moreover, active compounds like tannin may bind to the cuticle of the nematode, destabilize the membrane, and increase cell permeability by combining with membrane-associated sterols which lead to death [25].

Previous studies [21, 26] on the phytochemical analysis of *Lophira lanceolata* revealed the presence of flavonoids, saponins, polyphenols, tannins, anthraquinones, carbohydrates, glycosides, phenols, and free-reducing sugar. The ovicidal and larvicidal properties of *L*. *lanceolata* may be due to the presence of secondary metabolites, like alkaloids, saponins, polyphenols, carotenoids, tannins, coumarins, cardenolides, triterpenes, saponosides, embeline, and sesquiterpene lactones, which enter its phytochemical composition. Other researchers including [12, 13, 22] have shown that carotenoids, triterpenes, saponins, steroids, coumarins, tannins, and other chemical compounds of plants, like glycosides, enzymes, anthraquinones, essential oils, lipids, proteins, and fibers, if present in a plant, imply that the plant has antihelmintic properties.

## 5. Conclusion

The results obtained in our study scientifically validate the use of *L*. *lanceolata* in fighting against helminthes in Cameroonian folk medicine. However, further studies to determine its safety in order to assess its toxicity are needed to establish it as an antiparasitic agent.

## Figures and Tables

**Table 1:** Phytochemical screening of *Lophira lanceolata* extracts.

Extracts	Phytochemical composition					
	Alkaloids	Sterols	Triterpenoids	Saponins	Anthocyanins	Anthraquinones
Ethanol	+	−	+	−	+	+
Aqueous	+	−	+	+	+	+

## Data Availability

All the data generated and analysed are included in this research article.
